# Profiling *Mannheimia haemolytica* infection in dairy calves using near infrared spectroscopy (NIRS) and multivariate analysis (MVA)

**DOI:** 10.1038/s41598-021-81032-x

**Published:** 2021-01-14

**Authors:** Mariana Santos-Rivera, Amelia Woolums, Merrilee Thoresen, Ellianna Blair, Victoria Jefferson, Florencia Meyer, Carrie K. Vance

**Affiliations:** 1 Department of Biochemistry, Molecular Biology, Entomology, and Plant Pathology, Mississippi State University, Starkville, MS 39762 USA; 2 College of Veterinary Medicine, Pathobiology and Population Medicine, Mississippi State University, Starkville, MS 39762 USA

**Keywords:** Near-infrared spectroscopy, Respiratory tract diseases

## Abstract

Bovine respiratory disease (BRD) linked with *Mannheimia haemolytica* is the principal cause of pneumonia in cattle. Diagnosis of BRD traditionally relies on visual assessment, which can be untimely, insensitive, and nonspecific leading to inadequate treatment and further spread of disease. Near Infrared Spectroscopy (NIRS) is a rapid acquisition vibrational spectroscopy that can profile changes in biofluids, and when used in combination with multivariate analysis, has potential for disease diagnosis. This study characterizes the NIR spectral profile of blood plasma from dairy calves infected with *M. haemolytica* and validates the spectral biochemistry using standardized clinical and hematological reference parameters. Blood samples were collected for four days prior to (baseline), and 23 days after, a controlled intrabronchial challenge. NIR spectral profiles of blood plasma discriminated and predicted Baseline and Infected states of animal disease progression with accuracy, sensitivity, and specificity ≥ 90% using PCA–LDA models. These results show that physiological and biochemical changes occurring in the bloodstream of dairy calves during *M. haemolytica* infection are reflected in the NIR spectral profiles, demonstrating the potential of NIRS as a diagnostic and monitoring tool of BRD over time.

## Introduction

Bovine Respiratory Disease (BRD) is a multi-factorial, multi-pathogen condition causing billion-dollar losses to the worldwide cattle industry^1–4^. BRD associated with *Mannheimia haemolytica,* a gram-negative coccobacillus that is a constituent of the normal flora of the upper respiratory system of ruminants^5^, is the principal cause of pneumonia in cattle^5,6^. After environmental stress or viral infection, suppression of the host’s defense mechanisms may occur, and the replication rate of *M. haemolytica* in the upper respiratory tract increases rapidly, followed by inhalation and colonization of the lungs^1^. It is during this growth phase that virulence factors, such as leukotoxin (LKT), lipopolysaccharide (LPS), bacterial capsule, adhesins, and neuraminidase, are released by *M. haemolytica* to elude the innate and adaptive immune responses, allowing it to inhabit the lungs and establish infection^6,7^. Once a substantial level of infection occurs, a progression of observable symptoms in the host follows, beginning with a loss of appetite and is followed by depression, an increase in mucopurulent ocular and nasal secretions, fever (up to 42 °C), moist coughing, and a rapid, shallow respiratory rate. Clinical exam by auscultation of the cranioventral lung field typically reveals increased bronchial sounds, crackles, and wheezes. In severe cases, pleuritis may develop, characterized by an irregular breathing pattern and grunting on expiration^5,7,8^.

Visual-clinical diagnosis (VCD) of cattle appearance and behavior, after signs of infection are presented, is currently the standard approach to detecting BRD. Unfortunately, asymptomatic or mildly symptomatic cattle in the early stages of the disease are not identified or treated appropriately with antibiotics, leading to the spread of the infection throughout the herd^8,9^. The estimated sensitivity of traditional VCD is only 62%, leaving 38% of cattle with BRD unidentified and untreated^10^. Similarly, with only a 63% specificity rate, uninfected cattle constitute 37% of all cattle that are treated for BRD^10–12^. While failure to identify and treat affected cattle leads to negative impacts on animal welfare and productivity, unnecessary treatment of healthy cattle leads to financial loss associated with wasted medication and increases the risk of inducing antibiotic resistance in exchangeable pathogenic bacteria^2,4,5^. Transthoracic ultrasound (TU) evaluation is a diagnostic technique used to identify BRD in young dairy calves on the farm, and in real time^13,14^. The sensitivity and the specificity of TU were reported to be 89% and 95%, respectively, and were validated by the presence of lung consolidation with ≥ 3 cm of depth caudal to the heart where active pneumonia can be detected, in addition to referencing against respiratory scores and haptoglobin levels (≥ 15 mg/dL) in the blood serum^15^. Haptoglobin is an acute phase response protein and an early indicator of inflammation and is measured by ELISA (Enzyme-Linked ImmunoSorbant Assay)^16–18^.

Other laboratory techniques under consideration for early BRD diagnosis include direct detection of the infectious agent by Polymerase Chain Reaction (PCR), metabolomics profiling using multichannel nuclear magnetic resonance (NMR), or volatile compound biomarker analysis by Gas chromatography-mass spectrometry (GC-MS)^8,19–22^. However, these approaches require substantial sample purification and preparation and thus are not only labor-intensive and time-consuming but require specialized facilities for housing sophisticated instrumentation. Furthermore, the special handling, storage, and transport of biological samples to such external laboratories increase the risk of contamination and degradation. Due to the remoteness of most farms, such analytical methodologies are not feasible for in-field or real-time applications. Since BRD associated with *M. haemolytica* poses a serious animal welfare and economic problem, a rapid, portable, and accurate diagnostic test that can be used under field conditions is needed to correctly identify cattle that may be asymptomatic or in the early stages of respiratory infection and thus facilitate proper treatment to mitigate disease spread.

Near-Infrared Spectroscopy (NIRS) employs photon energy (*hν*) in the wavelength range 750–2500 nm to excite vibrational modes of organic compounds^23^ and, depending on the application, requires minimal sample preparation. Historically, NIRS has been used in agriculture to quantify nutrient composition for crops and animal diets and in quality control across the food indudustry^24^. The speed, accuracy, and the new advancements in portable and handheld NIR spectrometers have elevated this analytical technique to applications in the pharmaceutical and medical industries^25–27^. NIRS, combined with chemometrics based multivariate analysis (MVA)^28^ and concepts developed in the emerging field of Aquaphotomics, may be able to identify and discriminate the biochemical profile of blood plasma associated with *M. haemolytica* infection in dairy calves. Aquaphotomics is a complementary subfield of NIRS focused on water bonding modes and development of spectral profiles for defined aqueous systems, with increasing applications for the analysis of biological fluids, such as blood plasma, serum, urine, and milk^29–32^. In biological systems such as blood plasma, although water is the solvent, its microspectrum is sensitive to changes in solute composition^31^. Besides, the strong NIRS absorbance of OH bonds in water is known to enhance the signal from other molecules in the solution^33^. To this point, in human blood plasma, NIRS has been used to successfully profile Human Immunodeficiency Virus Type-1 (HIV-1) infection^34,35^, Alzheimer’s disease^36–38^, and lactate content^39^. In ruminants, blood plasma has been evaluated using NIRS for early pregnancy diagnosis of sheep^40^.

Infectious agent challenge studies offer controlled, standardized conditions in order to follow infection progress and enabling direct comparison across disease stages^41^. The advantage of a clinical, hematological, and biochemical profile obtained through a controlled challenge that differentiates diseased and healthy individuals would represent a significant achievement in the evaluation and management of BRD. Thus, this study aims to identify and discriminate the clinical signs, blood parameters, and the NIRS spectra of blood plasma obtained from a controlled bacterial challenge, in order to profile responses of dairy calves to *M. haemolytica* infection using multivariate analysis. Our long-term goal is to create a diagnostic strategy for the detection and treatment of animals in the early stages of BRD, thereby contributing to the sustainability of the food supply chain.

## Materials and methods

### Bacteria preparation

In preparation for the controlled bacterial challenge, *Mannheimia haemolytica* isolate D153 was streaked onto BD Brain Heart Infusion (BHI) agar (Bacto 237,500) and incubated overnight at 37 °C. A single colony was utilized to inoculate a 5 mL BHI broth (Difco 241,830) starter culture, which was maintained overnight in a shaker incubator at 200 rpm at 37 °C, diluted 1:100 and incubated again overnight. On the day of the challenge, the culture was again diluted 1:100, and incubated at 200 rpm and 37 °C until Abs_600_ = 1.0, at which point bacteria were pelleted at 10,000 rpm for 10 min at 4 °C and then resuspended to a final concentration of 1.0 × 10^9^ colony-forming units cfu/30 mL in 0.9% saline. The challenge inoculum was administered at 1.0 × 10^9^ cfu for every 91 kg of calf body weight.

### Animals and *M. haemolytica* challenge

Five non-vaccinated and immunologically mature Holstein steers were housed at Mississippi State University (MSU) for the *M. haemolytica* challenge (Table 1). The animal experiments were carried out with the approval of the MSU-Institutional Animal Care and Use Committee and all methods were performed in accordance with MSU-IACUC guidelines and regulations (IACUC-19–037). The bacterial challenge was given via bronchoalveolar lavage (BAL) catheter. Briefly, each calf was physically restrained in a chute, and a halter and lead were used to position the calf’s head straight up. Five mL of 2% lidocaine was used as a local anesthetic and was squirted into one nostril, and the BAL tube was introduced through this nostril and gently advanced into the trachea until the end of the catheter wedged into a bronchus. The appropriate volume of challenge inoculum, based on body weight, was administered through the BAL catheter, followed immediately by 60 mL of sterile 0.9% saline and 120 mL of air. Following the challenge procedure, a sub-sample of the challenge inoculum was used to prepare a quantitative culture assay on BHI agar and was checked at 24–48 h, and cfu were counted at 96–120 h. VCD was performed by an experienced veterinarian, and Complete Blood Counts (CBC) were assessed pre- and post-challenge before blood sample collection during four baseline days, 11 days immediately after challenge, and then every other day until 23 days post-infection.

**Table 1:** Number of blood samples contributing to the databases collected before and after the *M. haemolytica* challenge.

Calf ID	Age (months)	Weight (kg)	Baseline samples	Infected samples	Total
1	6	204	4	3	7
2	6	196	4	11	15
3	6	145	4	3	7
4	5	124	4	3	7
5	5	142	4	3	7
Total samples			20	23	43
Total spectra			200	230	430

Calf 2 presented mild signs of the infection from Day 1 until Day 19 when his signs aggravated, and antibiotics were then provided.

### Blood collection

A total of 18 blood samples were collected for each calf during the 23 days of the study (n = 90). Blood samples were collected through jugular venipuncture into two commercial blood collection tubes containing the anticoagulant Ethylenediaminetetraacetic acid (EDTA) and immediately placed on ice. One tube was centrifuged at 4000 rpm for 20 min for plasma separation and stored in duplicates of 1 mL at − 80 °C until NIRS analysis. The second tube was used for CBC analysis using a veterinary hematology analyzer for hematocrit (HCT), hemoglobin (HGB), mean corpuscular hemoglobin (MCH), mean corpuscular hemoglobin concentration (MCHC), mean corpuscular volume (MCV), red blood cell count (RBC), red cell distribution width (RDW), white blood cell count (WBC), and platelets (PTLs). In addition, a microscopic differential count of nucleated cells was carried out to test the variability between neutrophils (polymorphonuclears, PMNs), eosinophils (polymorphonuclear eosinophils, PMEs), basophils (polymorphonuclear basophils, PMBs), monocytes (MOs), and lymphocytes (LYs).

### Statistical analysis for clinical and hematological profiles

The VCD included rectal temperature (TEMP) recorded with a digital thermometer, heart rate (HR), respiratory rate (RR), and assessment of overall airway health (the type of cough, secretions) conducted by an experienced veterinarian using a stethoscope. Using VCD and the antibiotic therapy provided when the clinical signs aggravated as the reference parameters, blood plasma samples (n = 90) were categorized as Baseline (n = 20), Infected (n = 23), Infected and Treated with antibiotics (n = 21), and Recovered (n = 26); only the first two categories (Baseline and Infected) were selected for the application of the statistical analyses (Table 1) to avoid the interference of the antibiotic effect in the biochemical profile. VCD and CBC information was analyzed using univariate statistics to obtain the Mean and the standard deviation (SD). Significance in parameter response was tested between the two categories Baseline and Infected, using ANOVA and a pairwise mean comparison applying Student’s t-test with alpha = 0.05 (JMP 14.0 SAS Institute Inc., NC. USA). In addition, Principal Component Analysis (PCA) was applied using full cross-validation, and algorithm-SVD (Singular Value Decomposition) in order to determine the influence of clinical signs and CBC values in the baseline and infected periods (Unscrambler X v. 10.5 software CAMO Analytics, Oslo, Norway).

### NIR spectral signature collection

Transmittance NIRS spectra (n = 430) were collected using a portable ASD FieldSpec 3 + IndicoPro (Malvern Panalytical, ASD Analytical Spectral Devices Inc. Boulder, CO. USA). Samples were thawed over ice for 15 min and warmed between hands for approximately 1 min before NIR spectra collection. Plasma samples (300 µl) were analyzed in a 1.00 mm quartz cuvette mounted in an ASD-fibre optic cuvette adapter. Each NIR spectrum was collected across the range 350–2500 nm (interval = 1.4 nm for the region 350–1000 nm and 2.0 nm for the region 1000–2500 nm; 50 scans; 34 ms integration). Prior to plasma spectra collection, a reference spectrum was captured from an empty cuvette. Ten independent spectral signatures were collected per sample, repacking the cuvette with plasma between each replicate.

### Multivariate analysis (MVA)

The chemometrics based MVA was carried out in Unscrambler X v.10.5 and performed on the first overtone region of the near-infrared spectrum in the vibrational combination band between 1300 and 1600 nm. The mathematical pre-treatments of Linear Baseline Correction, Standard Normal Variate (SNV) with de-trending (polynomial order: 2), and a 2nd derivative (symmetric Savitzky–Golay smoothing, points = 12) were applied to all the databases described next. A balanced database (n = 300) was created by randomly selecting 30 spectral signatures per calf and per each category (Baseline or Infected), to ensure the homogeneity of variance and weight of the datasets by controlling for the imbalance and diversity of the total number of blood samples collected (Table 1). This database (DB0) included spectra from all five calves and was used to perform the PCA and the Aquaphotomic analyses. To test for mathematical pre-processing and modeling bias, and against the null hypothesis (no biological signature can be differentiated between samples from these two classes) in the discriminant analysis, five datasets were created by stratified random sampling and analyzed in a leave-one–animal-out approach, Table 2. Specifically, spectra obtained from samples from four calves were sorted into an 80/20% distribution to form the calibration and internal validation sets; plasma spectra from the remaining calf were used as the external validation set.

**Table 2:** Balanced databases distribution of spectra collected before and after *M. haemolytica* challenge.

Calf ID	DB0		Process	DB1		DB2		DB3		DB4		DB5	
	B	I		B	I	B	I	B	I	B	I	B	I
1	30	30	CAL	External validation		24	24	24	24	24	24	24	24
			VAL			6	6	6	6	6	6	6	6
2	30	30	CAL	24	24	External validation		24	24	24	24	24	24
			VAL	6	6			6	6	6	6	6	6
3	30	30	CAL	24	24	24	24	External validation		24	24	24	24
			VAL	6	6	6	6			6	6	6	6
4	30	30	CAL	24	24	24	24	24	24	External validation		24	24
			VAL	6	6	6	6	6	6			6	6
5	30	30	CAL	24	24	24	24	24	24	24	24	External validation	
			VAL	6	6	6	6	6	6	6	6		
Total CAL				192		192		192		192		192	
Total VAL				48		48		48		48		48	
Total spectra				240		240		240		240		240	
External validation				40	30	40	110	40	30	40	30	40	30

Each database has spectra from one animal removed for external validation.

DB, Database; B, Baseline; I, Infected; CAL, Calibration; VAL, Internal Validation.

### Principal component analysis

PCA was applied to the DB0 database and to the calibration sets created for the discriminant analysis, it was completed as a first step to observe spectral features from both baseline and infected plasma samples to determine the dataset factorizations and scores distributions, identify dominant peaks in the loadings and outliers using the Hotelling’s T^2^ influence plot. PCA on the mean-centered matrix was obtained using full random cross-validation and algorithm-SVD according to Eq. (1) where T = Scores, P = Loadings, E = Residual, U = First left singular values, D = Singular values, and V = First right singular values^42^.

1 $$ X = TP^{T} + E = \left( {UD} \right)V^{T} + E $$

### Aquaphotomics

Aquaphotomics was applied as a complementary spectral analysis to distinguish the biochemical profile of blood plasma collected before and after the *M. haemolytica* challenge. Water microstructure is represented by 12 spectral bands in the first overtone of the OH stretching region between 1300 and 1600 nm; these Water Matrix Coordinates (WAMACS) were used to generate barcodes and aquagrams to distinguish NIR spectra of plasma from baseline and infected stages^29,30^. To generate the barcodes, which represent wavelength shifts, the mean spectral information from sterile distilled water was subtracted from the mean spectrum for each category (Baseline or Infected) obtained from the transformed balanced database DB0. Aquagrams emphasize changes in the magnitude of peaks and were created by comparing the mean normalized (SNV only) water absorbance spectral patterns (WASPs) of the blood plasma from baseline and infected stages of the *M. haemolytica* challenge.

### Linear discriminant analysis

Linear Discriminant Analysis (LDA) was used in the transformed spectra containing the water information (1300–1600 nm) from the balanced databases (DB1–DB5). Before the application of LDA for spectra classification between Baseline and Infected categories, the dimensionality of each spectral database was reduced using PCA to overcome the constraint of requiring more objects (samples) than features (scores or PCs). PCs or factors capturing ≥ 99% of the variance in the calibration datasets were selected to build the PCA–LDA model for each balanced database^28,43^. The subsequent LDA based on Bayes’ formula, identifies similar spectral features for intra-class groupings and differential spectral features to separate the classes of blood plasma collected before and after the *M. haemolytica* challenge^44^. The PCA–LDA for the five prediction models created from databases DB1-5 is reported from the confusion matrix as a percent (%) to describe the quality parameters of accuracy, sensitivity, and specificity to evaluate the performance of the classification method^45^. The sensitivity test quantifies the PCA–LDA model’s ability to correctly identify the true positives of *M. haemolytica* infection^46^ described by Eq. (2) where TP = True positive, and FN = False negative^45^. A sensitivity of 85% detects 85% of animals with the disease (true positives) but classifies 15% of infected animals as healthy (false negatives). A high sensitivity (≥ 90%) is essential where the prediction model is used to identify severe but treatable diseases^47^.

2 $$ Sensitivity\;\% = \left( {\frac{TP}{{TP + FN}}} \right) \times 100 $$

The specificity of the PCA–LDA model is the ability of the model to correctly identify uninfected subjects, or the true negatives, and is represented by Eq. (3), where TN = True negative, and FP = False positive^45^. For example, a specificity of 85% correctly reports 85% of true negatives, but 15% of uninfected animals will be incorrectly identified as false positives^47^.

3 $$ Specificity\;\% = \left( {\frac{TN}{{TN + FP}}} \right) \times 100 $$

The prediction equations from the five models were applied to the internal validation and external validation sets described in Table 2 and evaluated with the same quality parameters. PCA scores plots and PCA–LDA plots were visualized in JMP 14.0.

## Results

### Clinical and hematological profile for *M. haemolytica* infection

**Figure 1:**
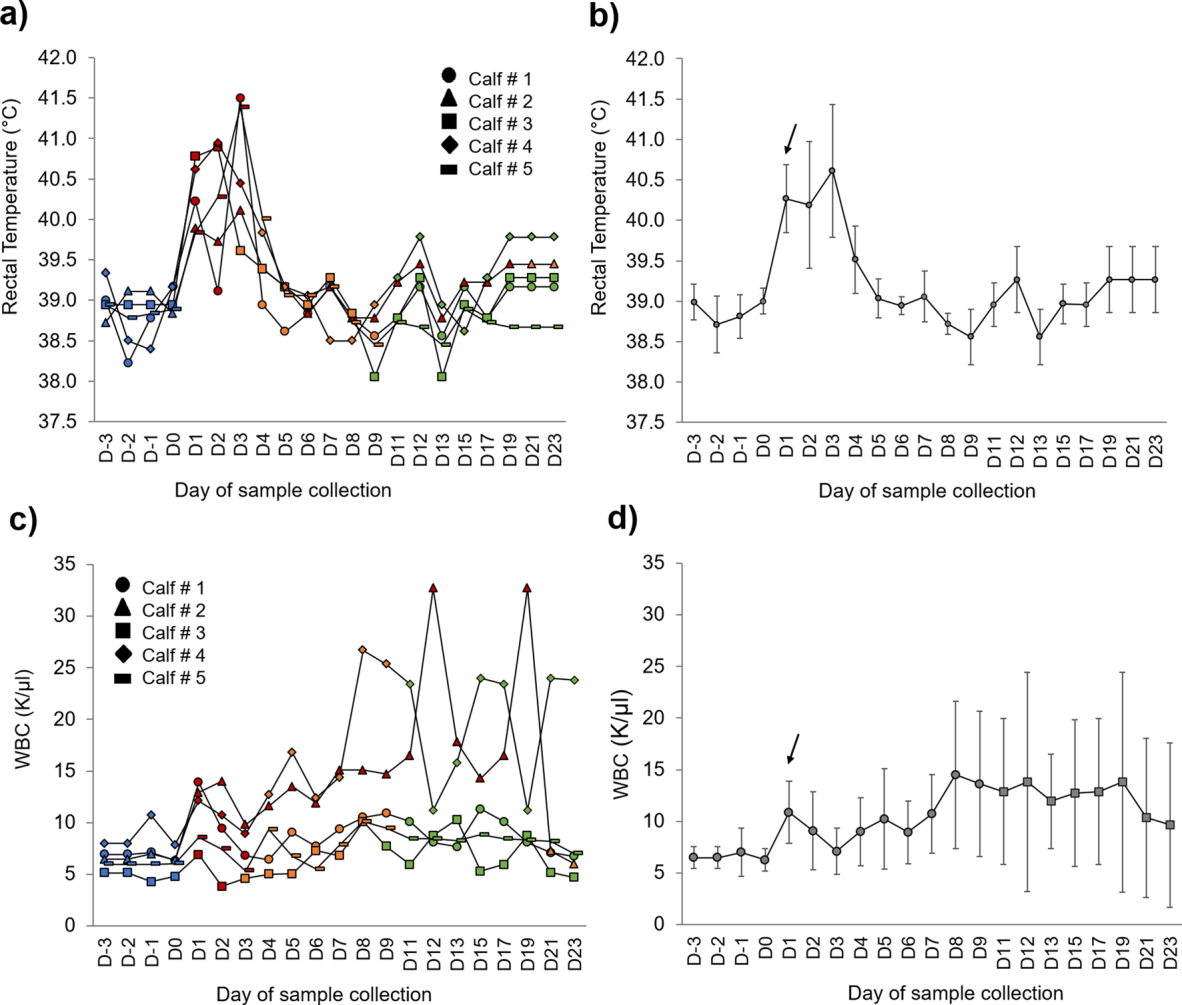
Dairy Calves (n = 5) response to *M. haemolytica* infection across the 23 days of the study*.* (**a**) Daily rectal temperature (°C) per calf. (**b**) Rectal temperature shown as Mean ± SD. The baseline days are in blue, infected days in dark red, the days of antibiotic treatment in orange, and the recovered days are in green. (**c**) White blood cell count (thousands per cubic milliliter, K/µl) per calf. (**d**) WBC showed as Mean ± SD. A characteristic increase 24 h after the challenge (D0, indicated by arrows) can be seen due to the activation of the innate immunity or nonspecific defense mechanisms against *M. haemolytica* virulence factors.

In terms of fever response and rectal temperature, all calves responded similarly before and after bacterial challenge on Day 0 (Fig. 1a). Due to severe disease on Day 3 post-challenge, four calves (ID 1, 3, 4, and 5) were treated over five days with a third-generation cephalosporin antibiotic with broad-spectrum activity (Ceftiofur), which caused the recovery of the calves and the change in the trends for TEMP and WBC (Fig. 1b). In the case of calf 2, mild signs of the infection were present from Day 1 until Day 19 when his signs aggravated, and antibiotics were provided. Only two calves, 2 and 4, exhibited a variable increasing pattern in WBC starting on Day 8, while the other three calves 1, 3, and 5 maintained pre-challenge levels of WBC due to the antibiotic therapy (Fig. 1c). When the rectal temperature and the WBC are averaged across individuals, a noticeable increase can be observed 24 h after the *M. haemolytica* challenge, which is characteristic of this particular bacterial infection in cattle (Fig. 1b, d).

The mean and standard deviations from the current variables used to identify *M. haemolytica* infection in the VCD and CBC and the bovine reference values for blood parameters^48^, are listed in Table 3. When taking into account only the baseline and infected stages of the disease in the controlled challenge, there was a significant (*p* < 0.05) increase in the rectal temperature, heart rate, respiratory rate, WBC, and the percentage PMNs following the challenge with *M. haemolytica.* By contrast, RBC and the percentage of lymphocytes decreased after infection compared to the Baseline category but remained in the range of the reference values.

**Table 3:** Clinical and hematological values (Mean ± SD) compared to reference for dairy calves challenged with *M. haemolytica*.

Analysis		Reference value	Baseline		Infected
**Visual-clinical diagnosis (VCD)**					
Temperature (°C)	TEMP	38.0–39.2	38.7 ± 0.5		39.8 ± 1.5*
Respiratory rate per minute	RR	26–50	35.8 ± 9.6		89.9 ± 19.2*
Heart rate per minute	HR	48–84	91.2 ± 10.2		101.0 ± 17.7*
**Complete blood counts (CBC)**					
Red blood cell count (M/µl)	RBC	5–11	7.8 ± 0.4*		7.3 ± 0.8
Hemoglobin (g/dl)	HGB	7.7–15.0	10.3 ± 0.5		9.9 ± 0.9
Hematocrit (%)	HCT	25–45	26.6 ± 5.9		27.1 ± 2.2
Platelets (K/µl)	PLTs	200–900	790.0 ± 189.4		778.0 ± 197.3
White blood cell count (K/µl)	WBC	4–12	6.6 ± 1.4		12.4 ± 5.5*
Neutrophils (%)	PMNs	27–72	29.5 ± 6.7		49.6 ± 17.7*
Lymphocytes (%)	LYs	22–64	41.7 ± 8.7*		27.7 ± 16.4
Eosinophils (%)	PMEs	0–12	1.1 ± 0.9		0.5 ± 0.9
Monocytes (%)	MOs	0–10	26.6 ± 9.5		21.5 ± 12.1
Basophils (%)	PMBs	0–3	1.3 ± 1.0		1.0 ± 1.5

Data were analyzed using Student’s t-test between the groups with an α < 0.05 delineating significant treatment effects (*). K/µl = thousands per cubic milliliter, M/µl = Millions per microliter, g/dl = grams per deciliter.

**Figure 2:**
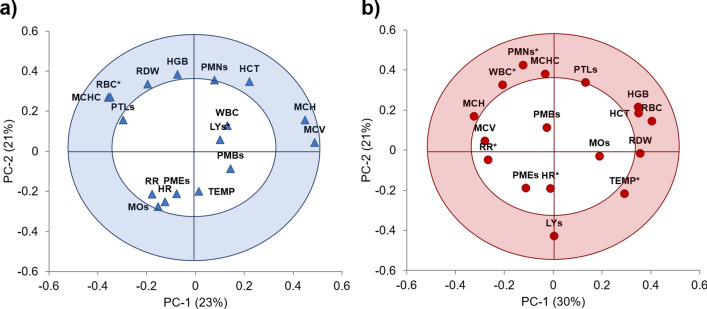
Visual-clinical diagnosis (VCD) and Complete blood counts (CBC) correlation loadings plots from the principal component analysis (PCA). The variables closest to the outer circle (shaded area) have the most influence in the variability of the database and are positively or negatively correlated with the disease stage of the dairy calves (n = 5); the points inside the inner circle are considered to have little or no influence. (**a**) Baseline or healthy calves; two PCs explained 44% of the variance, here as expected, the VCD variables and the white blood cell count (WBC) showed no influence. (**b**) Infected or challenged calves with *M. haemolytica*, two PCs explained 51% of the variation of the database. During this state of the disease the activation of the innate immunity showed a negative correlation in the rectal temperature (TEMP) and a positive correlation with the WBC and neutrophils (polymorphonuclears, PMNs).

The PCA correlation plots from VCD and CBC for each category (Baseline or Infected) are shown in Fig. 2. The first two PCs explained 43.6% and 50.4% of the variance of the VCD and CBC databases for the baseline and infected periods, respectively. No outliers were found in the Hotelling’s T^2^ influence plot (not shown). Prior to bacterial challenge, the parameters that are positively correlated and have the most influence indicating baseline levels were RBC, HCT, HGB, MCH, MCHC, RDW, and MCV (Fig. 2a). During the infection period, the activation of the innate immunity showed a negative correlation in TEMP and the percentage of LYs and a positive correlation with the WBC and the percentage of PMNs (Fig. 2b).

### Biochemical NIRS profile for *M. haemolytica* infection

In general, bovine blood plasma is made up of 92% water, 3% albumin and globulin, 4% immunoglobulins (α and β), 0.4% coagulants and fibrinogen, 0.5% minerals (sodium, potassium, bicarbonate, chloride, calcium), and 0.07% of lipids related with hormone content^49,50^. The mean raw and the transformed NIR spectral signatures from the bovine plasma collected before and after the *M. haemolytica* challenge displayed a characteristic and expected spectral water pattern in the wavelength range from 1300 to 1600 nm (Supplementary Fig. S1 online). This reflects the need for the application of Aquaphotomics and chemometric analyses to unveil the biochemical profile of the infection in this complex biofluid.

**Figure 3:**
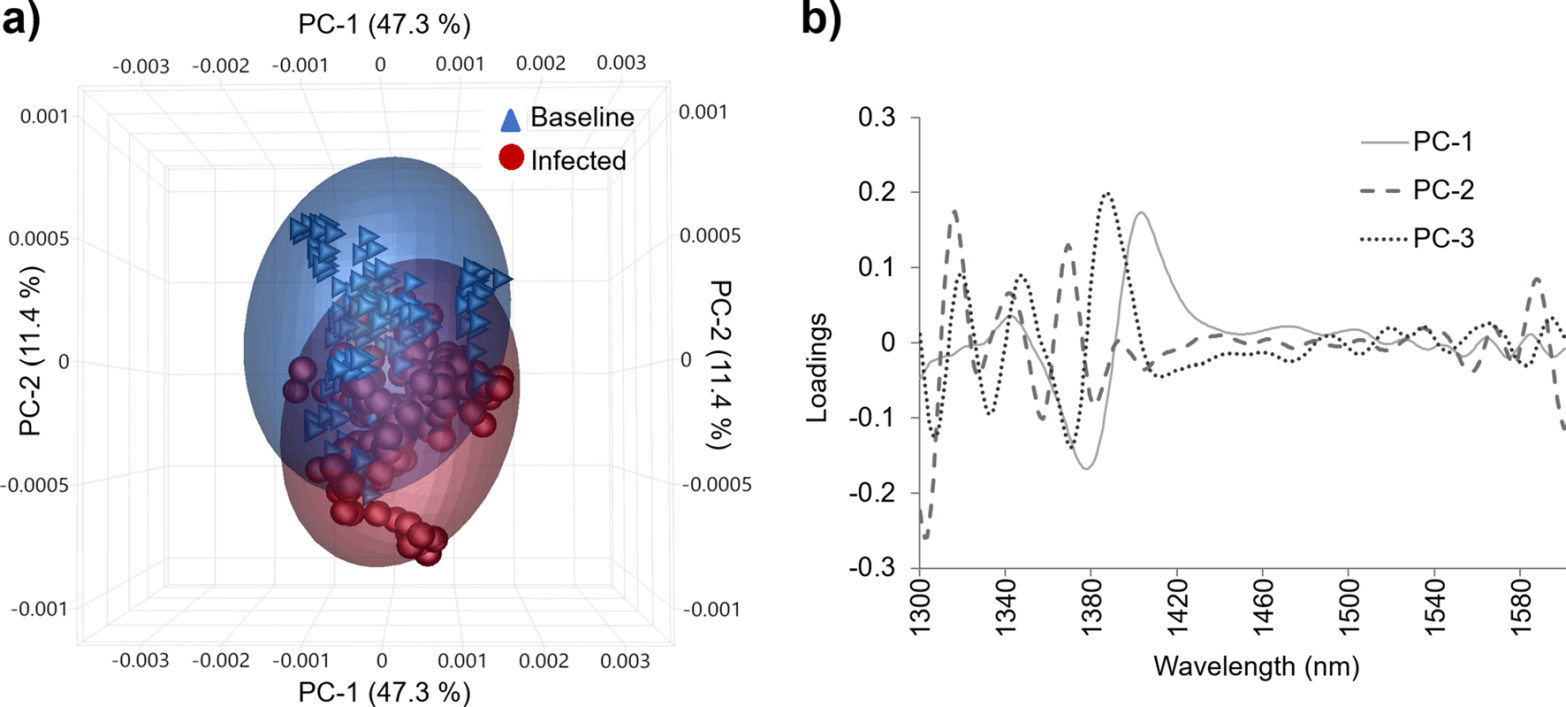
Principal component analysis (PCA) of the transformed blood plasma NIR spectra (1300–1600 nm) collected before and after *the M. haemolytica* challenge using the balanced spectral database DB0 (n = 300). (**a**) PCA scores plot for samples from the baseline and infected periods containing the scores from the first three PCs explaining 66.7% of the total variance. (**b**) PCA loadings showing the dominant peaks influencing the trends in the scores plot: PC-1 = 47.3%, PC-2 = 11.4%, PC-3 = 8.0%.

The complex chemical differences and similarities in the transformed spectra from bovine blood plasma between 1300 and 1600 nm where OH, CH, and NH bonds interact with NIR light^23^ can be observed in the trends from the three dimensional PCA scores plot (Fig. 3a). Here the first three PCs explained 66.7% of the variation of the DB0 database. No outliers were found in the Hotelling’s T^2^ influence plot (not shown), eliminating the possibility of artificial bias. The PC loadings show the dominant peaks influencing the trends in the scores plot (Fig. 3b). The first PC loading, PC-1 explained 47.3% of the variance of the database; PC-2 and PC-3 explained another 11.4% and 8.0% of the variance, respectively.

**Figure 4:**
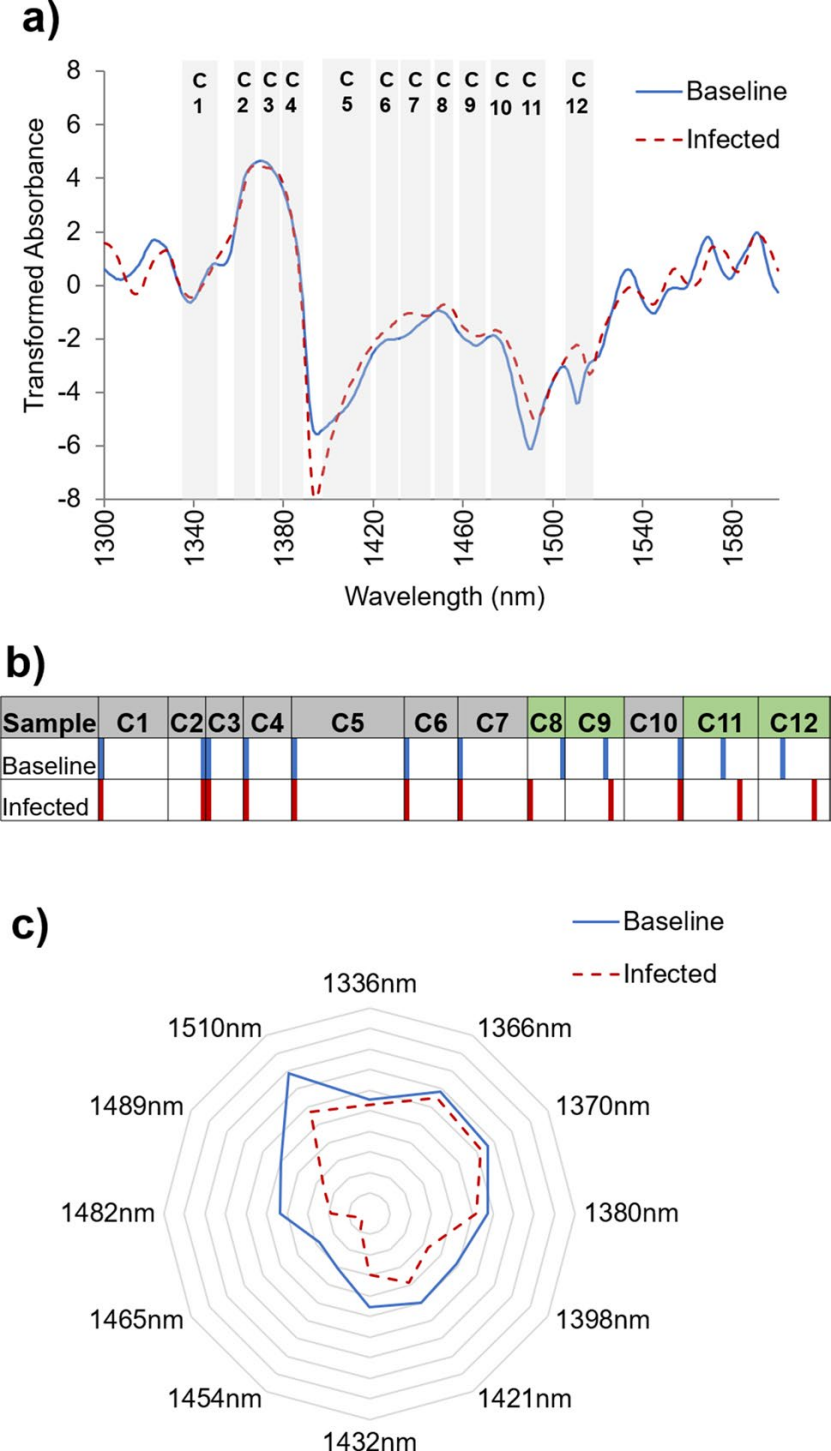
Aquaphotomics. (**a**) Transformed NIR spectra corresponding to bovine plasma collected before and after the *M. haemolytica* challenge*.* The spectral information from sterile distilled water was removed from the average spectra. (**b**) Peak shifts can be observed in the WAMACS barcode in the coordinates C8, C9, C11, and C12 for the baseline and infected blood plasma. (**c**) Aquagram displaying the normalized averaged spectra (SNV only) showing different WASPs. The highest points of absorbance were found at 1366 and 1510 nm for the Infected and Baseline plasma, respectively.

The complementary Aquaphotomic analysis displayed a different spectral pattern for the Baseline and Infected blood plasma in the wavelength range 1300–1600 nm (Fig. 4a). Barcodes highlight a shift in the WAMACS^31^ for the baseline and infected blood plasma samples, reflecting changes in the plasma constituents and the water matrix. In the coordinate C8 (1448–1454 nm), a peak shift to higher frequency can be observed in the infected blood plasma spectra to 1448 nm in comparison with the baseline peak at 1454 nm, indicating a shift from bulk water dynamics to an increase in the formation of complex three-dimensional molecular spheres or hydration shells around solute molecules^31,51^. Additionally, in the spectra collected from blood plasma collected during the infected stage, NIR spectral peaks are right-shifted in C9 (1458–1468 nm), C11 (1482–1495 nm), and C12 (1506–1516 nm), more specifically to1466, 1492 and 1516 nm in comparison with spectral peaks at 1465, 1489, and 1510 nm from the blood plasma collected during the baseline stage; suggesting a shift towards more strongly bound water and longer-lived water complexes^52^. More highly organized water complexes and limited water exchange from solvation shells to bulk water occurs as solubility decreases, either in the context of concentration or reduced polarity of solutes (Fig. 4b).

The aquagram was created with the highest absorbances from the WAMACS in the baseline plasma at 1336, 1366, 1370, 1380, 1398, 1421, 1432, 1454, 1465, 1482, 1489, and 1510 nm (Fig. 4c). Overall, different WASPs can be observed for the blood plasma collected before and after the *M. haemolytica* challenge. Higher absorbances at 1366 and 1510 nm for the Infected and Baseline plasma are observed. These spectral absorbance points belong to the coordinates C2 (1360–1366 nm) and C12 (1370–1376 nm), and represent a higher number of strongly bound water molecules to non-water constituent molecules in the baseline period, and molecules organized in water symmetrical and asymmetrical stretching vibrations associated with changes in the solute composition of the blood plasma during the infection period^31^.

### Classification of the biochemical NIRS profile during *M. haemolytica* infection

**Figure 5:**
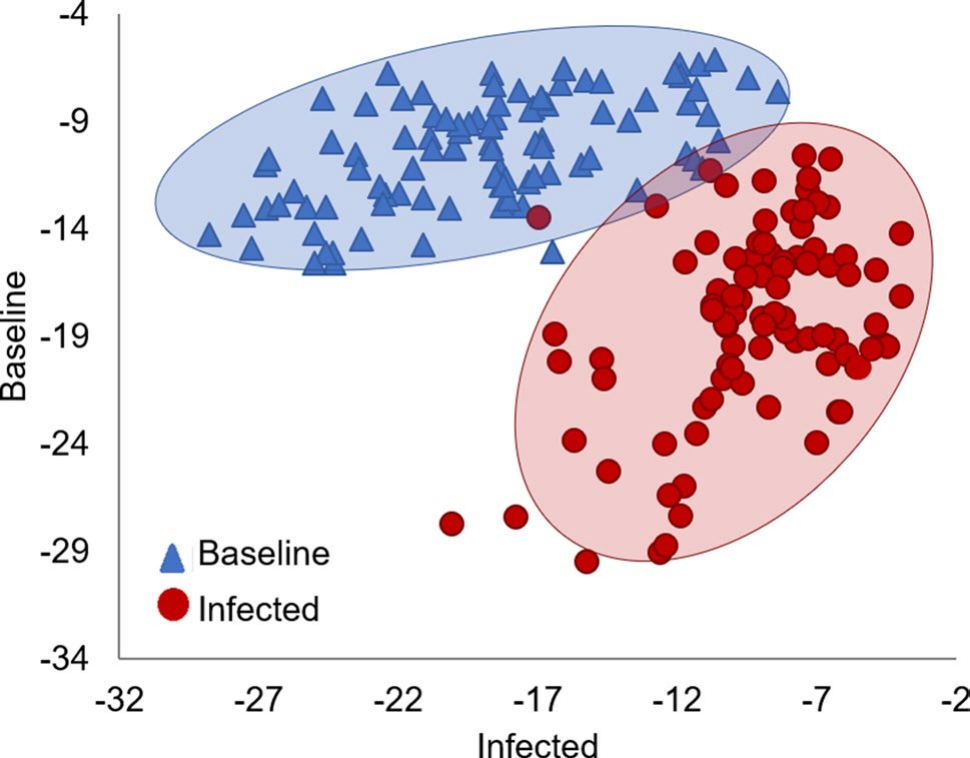
PCA–LDA plot for the calibration database DB1 excluding calf 1. Baseline = 100%, Infected = 97.9% of accurately classified spectra from bovine blood plasma collected before and after the *M. haemolytica* challenge.

The PCA–LDA was conducted on the spectra of plasma from both baseline and infected states simultaneously. A total of 18 PCs explaining 99 ± 0.2% of the variance in the PCA of the calibration sets were selected for the creation of the discriminant models using databases DB1 to DB5 (Supplementary Table S1 online). The derived prediction equations were then applied to the internal and external validation sets of spectra (Table 4). On average, the calibration of this supervised pattern recognition approach gave accuracy, sensitivity, and specificity of 98.5 ± 0.9, 97.5 ± 1.2, and 99.6 ± 0.9%, respectively. These values indicate that only 2.5 ± 1.2% of the infected blood plasma spectra were classified as false negatives, and 0.4 ± 0.9% of the baseline spectra corresponded to false positives to *M. haemolytica* infection. All the calibration databases (DB1–DB5) displayed similar trends in the PCA–LDA plot where two defined groups are observed; in Fig. 5, database DB1, excluding calf 1, is shown as a representation of the five databases analyzed. In addition, the internal validation set exhibited average values of 94.2 ± 1.7, 91.7 ± 2. 9, and 96.7 ± 1.9% for the accuracy, sensitivity, and specificity, respectively. In this process, 8.3 ± 2. 9% and 3.3 ± 1.9% of the spectra were classified as false negatives and false positives to the bacteria infection, correspondingly.

**Table 4:** PCA–LDA spectra classification and quality parameters for bovine plasma collected before and after the *M. haemolytica* challenge.

Database	Category and quality	%PCA–LDA		
		Cal 80%	Val 20%	External validation
DB1	Baseline	96/96	24/24	30/40
	Infected	94/96	22/24	30/30
	% Accuracy	99.0	95.8	87.5
	% Sensitivity	97.9	91.7	100
	% Specificity	100	100	75.0
DB2	Baseline	96/96	23/24	36/40
	Infected	95/96	22/24	69/110
	% Accuracy	99.5	93.8	76.4
	% Sensitivity	99.0	91.7	62.7
	% Specificity	100	95.8	90.0
DB3	Baseline	96/96	23/24	40/40
	Infected	92/96	23/24	24/30
	% Accuracy	97.9	95.8	90.0
	% Sensitivity	95.8	95.8	80.0
	% Specificity	100	95.8	100
DB4	Baseline	94/96	23/24	31/40
	Infected	93/96	22/24	30/30
	% Accuracy	97.4	93.8	88.8
	% Sensitivity	96.9	91.7	100
	% Specificity	97.9	95.8	77.5
DB5	Baseline	96/96	23/24	38/40
	Infected	94/96	21/24	16/30
	% Accuracy	99.0	91.7	74.2
	% Sensitivity	97.9	87.5	53.3
	% Specificity	100	95.8	95.0
% Accuracy Mean ± SD		98.5 ± 0.9	94.2 ± 1.7	83.4 ± 7.5
% Sensitivity Mean ± SD		97.5 ± 1.2	91.7 ± 2.9	79.2 ± 21.3
% Specificity Mean ± SD		99.6 ± 0.9	96.7 ± 1.9	87.5 ± 10.9

Furthermore, the external validation set containing the spectra from the calf excluded during the calibration process was accurately classified with an average percentage of 83.3 ± 7.5%, a sensitivity of 79.2 ± 21.3%, and a specificity of 87.5 ± 10.9% when applying the prediction model. This indicates 20.8 ± 21.3% and 12.5 ± 10.9% of the spectra from the excluded calf to be classified as false negatives and false positives to *M. haemolytica* infection, respectively, when VCD is used as the reference method. These results suggest that the biochemical changes in the bloodstream as a result of the calves’ response to the bacterial infection have the potential to be accurately detected and classified using NIR spectroscopy.

## Discussion

The trends in the clinical and hematological parameters observed in our controlled challenge are related to the physiological response to *M. haemolytica* virulence factors associated with pathogenesis and needed to overcome the innate and adaptive immune response from the host. Our results are in line with similar profiles documented in previous studies when calves were challenged with either *M. haemolytica* alone or after they were exposed to Bovine Viral Diarrhea Virus Type 1B^8,53,54^. After the bacterial challenge, adherence and invasion of the respiratory tract cells occurs in response to the bacterial capsule, adhesins, and neuraminidase^5^. Once the host’s cells are infected, the LPS complex and other pyrogenic features of *M. haemolytica* are released, activating the innate immunity or nonspecific defense mechanisms, triggering the observed fever and an increase in phagocytic cells (macrophages and neutrophils) after 24 h of the infection^8,53^. Subsequently, adaptive immunity is affected, which is reflected by the decrease in lymphocytes due to LKT release by the pathogen. The interaction of LKT with the CD18 subunit of the β2 integrin receptor of leukocytes forms transmembrane pores causing leakage of oxygen radicals and other products, such as nitric oxide, lysosomal enzymes, and inflammatory mediators such as cytokines IL-1β and TNFα, into the surrounding pulmonary parenchyma^7^. This contributes to cell apoptosis and pulmonary necrosis associated with the irregular breathing pattern, grunting on expiration, increased bronchial sounds, crackles, and wheezes^5,7^. The differences found in the univariate and multivariate results here demonstrate physiological and hematological changes occurred in response to the *M. haemolytica* challenge. More importantly, these parameters reflect a characteristic physiological response of disease state and provide the basis of the NIR spectral profiles used to discriminate healthy from infected calves, which will be useful for future diagnosis and monitoring of infection over time.

The NIR spectral profiles obtained in the chemometrics based MVA and the complementary Aquaphotomics, indicate changes in the biochemical make-up of the bovine blood plasma between the baseline and infected stages of the *M. haemolytica* challenge reflecting a calf’s immune response to the infection through changes in inflammatory cellular energy metabolism and antimicrobial pathways^55^. The recruitment of inflammatory cells results in a shift in their energy demand such that the supply almost exclusively originates from glycolysis, in order to accomplish the processes of phagocytosis to promote microbial death^55,56^. At the same time, increased flux through the pentose phosphate pathway (PPP) occurs, generating the reducing equivalent NADPH^55^. When glucose becomes limiting, T-cells utilize alternative energy sources, such as glutamine (critical for nitrogen metabolism and protein transamination), or fatty acids which feed the tricarboxylic acid cycle (TCA) generating intermediates such as citrate and the reducing agent NADH for the electron transport chain and aerobic metabolism^56^. The NADPH and NADH resulting from the PPP and TCA cycle, respectively, are required to produce reactive oxygen species (ROS) by NADPH oxidase in the membrane of activated macrophages, which are the crucial mechanisms for killing phagocytosed bacteria^55^. Additionally, nitric oxide (NO) and downstream reactive nitrogen species (RNS) produced by NO synthase in activated macrophages exert microbicidal or microbiostatic activity against bacteria by using L-arginine imported into the cell for the synthesis of NO as an antimicrobial product^55^. The increase of energy substrates, products, and key metabolites from the metabolic shifts occurring in the host as a response to *M. haemolytica* infection provide a changing milieu of components contributing to the biochemical NIR spectral profile. In this way, NIR spectra obtained from the blood plasma collected from the dairy calves before and after the *M. haemolytica* challenge differ, thus providing the information needed for spectra classification representing disease state.

Using NIRS for determining infection from *M. haemolytica* in dairy calves has a threshold of 90% sensitivity and specificity within the calibration and validation spectra, which is comparable to other techniques currently considered for BRD diagnosis. For example, ELISA determination of serum haptoglobin has a sensitivity of 93% and a specificity of 86% using a cutoff of ≥ 0.81 mg/mL^8^. Likewise, triplex real-time PCR based on the V3/V4 region of the 16S rRNA gene of three Mycoplasma species (*M. bovis, M. bovirhinis, and M. dispar*), is reported to have a diagnostic specificity of 98.2, 99.1, and 100%, respectively, when using bronchoalveolar lavage fluid (BALF) from infected calves^19^. Moreover, blood plasma from cattle with or without signs of BRD was evaluated with NMR for metabolite identification and diagnosis using visual clinical diagnosis as the reference method. In this case, animals with signs of BRD demonstrated increases in plasma α-glucose chains, hydroxybutyrate, and phenylalanine, and decreases with tyrosine, glutamine, citrate, and glutamate compared to healthy control animals. The accuracy, sensitivity, and specificity in this NMR study were 93, 99, and 88% for the calibration and 81, 84, and 74% for the validation process, respectively^20^.

Blood plasma spectra containing water information was used for the discriminant analysis (PCA–LDA) of baseline and infected samples to include the biochemical information of a fluid imbalance that may occur during the bacterial infection, affecting the blood volume and the blood pressure and consequently, the blood plasma composition^57^. Our results for *M. haemolytica* infection in dairy calves are comparable with previous studies where human blood plasma was evaluated using NIRS to profile, detect, and classify diseases. In one study, PCA and soft independent modeling of class analogy (SIMCA) were applied to identify features of NIR spectra of plasma from patients infected with HIV-1 in comparison with healthy individuals^35^. Both the PCA scores plot and the SIMCA Cooman’s plot suggested that HIV-1 infections caused specific blood plasma changes and affect its Vis–NIR spectra from 600 to 1100 nm^35^. In other studies, infrared spectroscopy analysis of human blood plasma has been performed to discriminate mild, moderate, and severe cases of Alzheimer’s disease (AD), in comparison with normal elderly persons used as a control^36–38^. In one study, NIR spectra in combination with regression analysis based on an oxidative stress index for group classification of AD patients, reported an 80% sensitivity and 77% specificity to discriminate patients with this disease^38^. Similarly, NIR spectra in combination with PCA and quadratic discriminant analysis (PCA–QDA) displayed 92.8% accuracy, 87.5% sensitivity and 96.1% specificity to classify blood plasma from patients at various stages of AD^36^. In a different study, Fourier transform infrared (FT-IR) spectra were discriminated with a sensitivity of 89% and specificity of 92% for the presence of Alzheimer’s disease in blood plasma^37^.

In ruminants, blood plasma has been evaluated using NIRS for early diagnosis of pregnancy in sheep, discerning spectra from pregnant and non-pregnant females by partial least squares discriminant analysis (PLS-DA), and using ELISA for pregnancy-associated glycoprotein (PAG), progesterone (P_4_), and abdominal ultrasonography at 45 days after artificial insemination (AI) as reference methods. At Day 18 after AI, the sensitivity and specificity of NIRS and P_4_ for pregnancy detection were 98.9% and 100%, respectively. Likewise, on Day 25, these parameters were 100% for NIRS and PAG^40^. All these studies suggest that NIRS can be an accurate method of diagnosis with similar sensitivities and specificities as the reference methods used to analyze blood plasma.

## Conclusion

Clinical and hematological profiles for *M. haemolytica* infection were assessed using MVA, identifying the most reliable indicators of BRD in cattle among the numerous data parameters usually collected when disease is suspected. Based on these reference parameters and the associated biochemical changes occurring with the immune response, NIR spectral profiles were established for blood plasma collected before and after *M. haemolytica* infection. The NIR spectral profiles were used to develop a PCA–LDA model for discrimination of blood samples that can identify cattle infected with *M. haemolytica* with an accuracy, sensitivity, and specificity of > 90% within the model. Model prediction had an accuracy of 83.3 ± 7.5%, a sensitivity of 79.2 ± 21.3%, and a specificity of 87.5 ± 10.9% for NIR spectra of blood plasma collected from different animals than used in the model structure. Our data indicate NIRS is a promising approach to rapid in-line diagnosis of BRD caused by *M. haemolytica*.

## Supplementary information

Supplementary Information 1.

## Data Availability

Data is available upon request from Carrie K. Vance (ckv7@msstate.edu).
