# Supplementary material for: Profiling Mannheimia haemolytica infection in dairy calves using near infrared spectroscopy (NIRS) and multivariate analysis (MVA)
Source: Sci Rep. 2021 Jan 14;11:1392. doi: 10.1038/s41598-021-81032-x (PMC7809125; doi:10.1038/s41598-021-81032-x)
Supplement: Supplementary file 1 — Supplementary Information 1. [file 41598_2021_81032_MOESM1_ESM.docx]

**Profiling *Mannheimia haemolytica* infection in dairy calves using Near Infrared Spectroscopy (NIRS) and Multivariate Analysis (MVA)**

**Mariana Santos-Rivera^1^, Amelia Woolums^2^, Merrilee Thoresen^2^, Elliana Blair^1^, Victoria Jefferson^1^, Florencia Meyer^1^, and Carrie K. Vance^1*^**

^1^ Mississippi State University, Department of Biochemistry, Molecular Biology, Entomology, and Plant Pathology, Mississippi State, MS, USA 39762

^2^ Mississippi State University, College of Veterinary Medicine, Pathobiology & Population Medicine, Mississippi State, MS, USA 39762


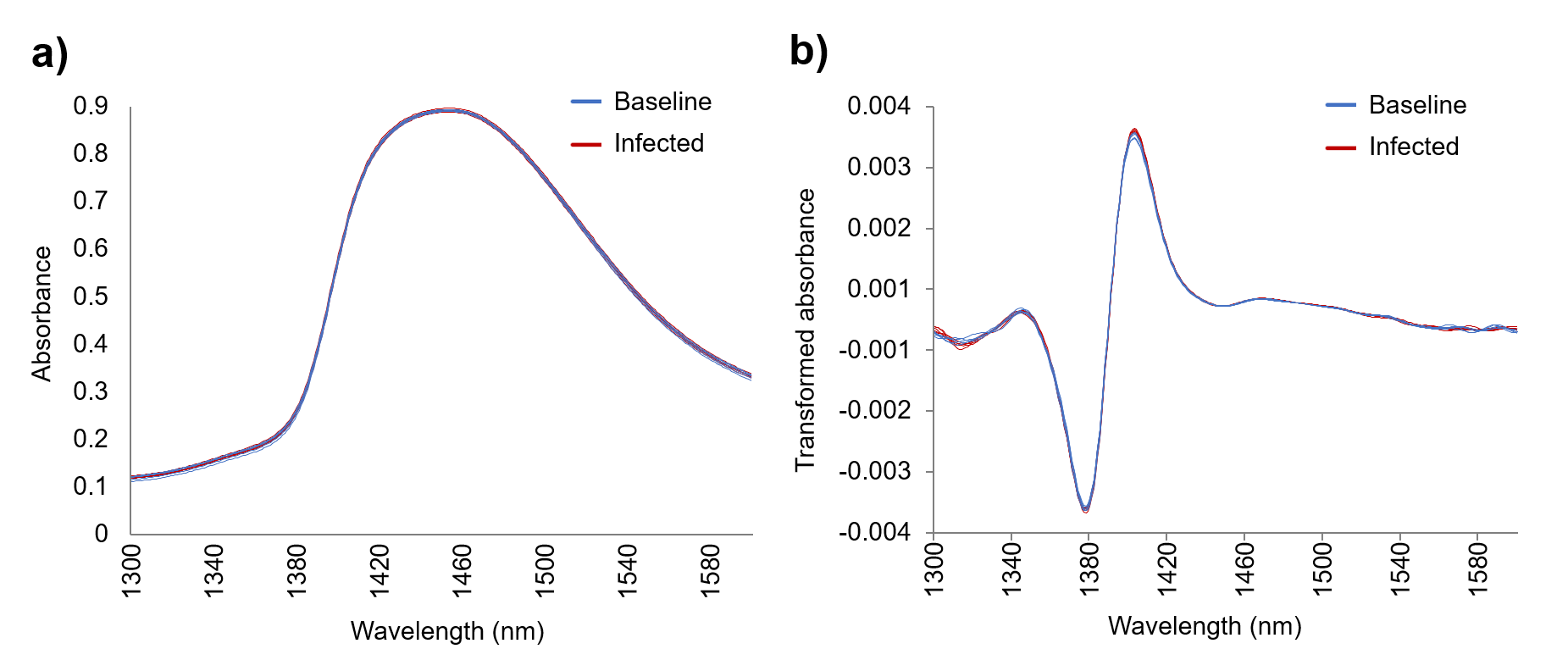


**Fig. S1.** Bovine blood plasma near-infrared spectra average (first overtone region of the NIR spectrum in the vibrational combination band between 1300-1600 nm) collected from each calf (n = 5) before and after *the M. haemolytica* challenge. (**a**) Raw or unprocessed NIR spectral signatures are showing the characteristic water spectral pattern. (**b**) Transformed or processed spectra shows two prominent features at 1378 and 1401 nm and the similarities between baseline and infected samples, reflecting the need for the application of Aquaphotomics and chemometrics based MVA to unveil the biochemical profile of the infection in this complex biofluid.

| **PCs** | **DB1 CAL** | | **DB2 CAL** | | **DB3 CAL** | | **DB4 CAL** | | **DB5 CAL** | | **Mean ± SD** | |
| --- | --- | --- | --- | --- | --- | --- | --- | --- | --- | --- | --- | --- |
|  | **PCs** | **LDs** | **PCs** | **LDs** | **PCs** | **LDs** | **PCs** | **LDs** | **PCs** | **LDs** | **PCs** | **LDs** |
| PC-1 | 45 | 45 | 50 | 50 | 49 | 49 | 45 | 45 | 46 | 46 | 47 ± 2.3 | 47 ± 2.3 |
| PC-2 | 55 | 10 | 62 | 12 | 60 | 11 | 59 | 14 | 57 | 12 | 59 ± 2.6 | 12 ± 1.5 |
| PC-3 | 65 | 10 | 68 | 6 | 69 | 9 | 67 | 9 | 66 | 9 | 67 ± 1.8 | 9 ± 1.5 |
| PC-4 | 71 | 7 | 74 | 6 | 75 | 6 | 74 | 6 | 73 | 7 | 73 ± 1.5 | 6 ± 0.5 |
| PC-5 | 77 | 6 | 80 | 5 | 80 | 5 | 78 | 5 | 78 | 5 | 79 ± 1.2 | 5 ± 0.4 |
| PC-6 | 82 | 5 | 85 | 5 | 84 | 4 | 83 | 4 | 83 | 5 | 83 ± 1.0 | 5 ± 0.5 |
| PC-7 | 86 | 4 | 88 | 3 | 87 | 3 | 86 | 3 | 86 | 3 | 87 ± 0.8 | 3 ± 0.4 |
| PC-8 | 88 | 2 | 90 | 2 | 89 | 2 | 88 | 2 | 89 | 2 | 89 ± 0.8 | 2 ± 0.0 |
| PC-9 | 90 | 2 | 92 | 2 | 91 | 2 | 91 | 2 | 91 | 2 | 91 ± 0.7 | 2 ± 0.0 |
| PC-10 | 92 | 2 | 94 | 2 | 93 | 2 | 92 | 2 | 93 | 2 | 93 ± 0.7 | 2 ± 0.0 |
| PC-11 | 94 | 1 | 95 | 2 | 94 | 1 | 94 | 1 | 94 | 1 | 94 ± 0.6 | 1 ± 0.4 |
| PC-12 | 95 | 1 | 96 | 1 | 95 | 1 | 95 | 1 | 95 | 1 | 95 ± 0.6 | 1 ± 0.0 |
| PC-13 | 96 | 1 | 97 | 1 | 96 | 1 | 96 | 1 | 96 | 1 | 96 ± 0.4 | 1 ± 0.0 |
| PC-14 | 97 | 1 | 98 | 1 | 97 | 1 | 97 | 1 | 97 | 1 | 97 ± 0.4 | 1 ± 0.0 |
| PC-15 | 97 | 1 | 98 | 0 | 98 | 1 | 98 | 1 | 98 | 1 | 98 ± 0.3 | 1 ± 0.4 |
| PC-16 | 98 | 1 | 98 | 0 | 98 | 1 | 98 | 1 | 98 | 0 | 98 ± 0.2 | 1 ± 0.5 |
| PC-17 | 98 | 0 | 99 | 0 | 98 | 0 | 99 | 1 | 98 | 0 | 99 ± 0.2 | 0 ± 0.4 |
| PC-18 | 99 | 0 | 99 | 0 | 99 | 0 | 99 | 0 | 99 | 0 | 99 ± 0.2 | 0 ± 0.0 |
| **Cumulative total** | **99** | **99** | **99** | **98** | **99** | **99** | **99** | **99** | **99** | **98** | **99 ± 0.2** | **99 ± 0.5** |

**Table S1.** Percentage (%) of explained variance from the PCA applied to the calibrations sets (balanced databases DB1 to DB5) used in the creation of the discriminant models. DB = Database, CAL = Calibration, PCs = Principal Components, LDs = Loadings, SD = Standard deviation.
